# Reading in the brain of children and adults: A meta‐analysis of 40 functional magnetic resonance imaging studies

**DOI:** 10.1002/hbm.22749

**Published:** 2015-01-27

**Authors:** Anna Martin, Matthias Schurz, Martin Kronbichler, Fabio Richlan

**Affiliations:** ^1^ Centre for Cognitive Neuroscience University of Salzburg Hellbrunnerstr. 34 5020 Salzburg Austria; ^2^ Neuroscience Institute, Christian Doppler Clinic, Paracelsus Medical University Ignaz‐Harrer‐Str. 79 5020 Salzburg Austria

**Keywords:** reading, development, child, adult, functional magnetic resonance imaging, neuroimaging, brain mapping, meta‐analysis

## Abstract

We used quantitative, coordinate‐based meta‐analysis to objectively synthesize age‐related commonalities and differences in brain activation patterns reported in 40 functional magnetic resonance imaging (fMRI) studies of reading in children and adults. Twenty fMRI studies with adults (age means: 23–34 years) were matched to 20 studies with children (age means: 7–12 years). The separate meta‐analyses of these two sets showed a pattern of reading‐related brain activation common to children and adults in left ventral occipito‐temporal (OT), inferior frontal, and posterior parietal regions. The direct statistical comparison between the two meta‐analytic maps of children and adults revealed higher convergence in studies with children in left superior temporal and bilateral supplementary motor regions. In contrast, higher convergence in studies with adults was identified in bilateral posterior OT/cerebellar and left dorsal precentral regions. The results are discussed in relation to current neuroanatomical models of reading and tentative functional interpretations of reading‐related activation clusters in children and adults are provided. *Hum Brain Mapp 36:1963–1981, 2015*. © **2015 The Authors Human Brain Mapping Published by Wiley Periodicals, Inc.**.

## INTRODUCTION

Learning to read requires the development of a highly organized brain system capable of integrating orthographic, phonological, and lexico‐semantic features of written words [Sandak et al., 2004]. According to the classical dorsal/ventral functional neuroanatomical model of visual word recognition [e.g., Pugh et al., 2000], the cortical system underlying skilled reading includes three functionally specialized regions contributing to different aspects of reading. (i) A left dorsal temporo‐parietal (TP) circuit around the classically termed Wernicke's area including the posterior superior temporal gyrus (STG) and supramarginal (SMG) and angular gyri (ANG) of the inferior parietal lobule (IPL). This dorsal system is associated with phonology‐based reading processes (i.e., grapheme–phoneme conversion, phonological assembly). (ii) A left ventral occipito‐temporal (OT) circuit including lateral extrastriate, fusiform (FFG), and inferior temporal (ITG) regions hosting the putative visual word form area (VWFA). This ventral system is linked to memory‐based visual‐orthographic word recognition. (iii) A left inferior frontal circuit around the classically termed Broca's area including inferior frontal (IFG) and precentral gyri (PRG). This anterior system is thought to be involved in speech‐gestural articulatory recoding of print.

According to the primary developmental assumption of this model, beginning readers primarily rely on the left dorsal TP circuit involved in phonology‐based reading via serial grapheme–phoneme conversion. Conversely, skilled and efficient readers engage the left ventral OT circuit for fast and automatic visual‐orthographic whole‐word recognition. In line with this assumption, Shaywitz et al. [2007] reported an age‐related increase in activation during a nonword rhyme judgment task in the left OT cortex. Furthermore, Turkeltaub et al. [2003] reported engagement of left posterior STG regions during an implicit word reading task early in the course of reading acquisition. They did not find, however, an increase in left OT activation with increasing age. Instead, bilateral OT regions were active in children and adults during both the implicit word reading task and a corresponding task with false font strings. In the OT cortex, the crucial developmental finding was a decrease in right hemisphere activation with increasing age. A similar developmental pattern of decreasing right extrastriate activation was reported by Brown et al. [2005]. Both of these studies, together with four other studies [Bitan et al., 2007b; Booth et al., 2003; Schlaggar et al., 2002; Shaywitz et al., 2002], reported an age‐related increase in activation in left IFG and PRG regions.

Interestingly, several studies provided evidence suggesting that the regions activated during reading in children are already as spatially restricted and lateralized as in adults [Booth et al., 2001, 2003; Brem et al., 2010; Church et al., 2008; Gaillard et al., 2003]. For example, Gaillard et al. [2003] reported marked activation during a silent reading task in left OT, middle temporal (MTG), IFG, and supplementary motor area (SMA) regions in children as young as 7 years. Similarly, using an overt word reading task, Church et al. [2008] found activation patterns in children and adults to be similar in location and laterality. Although some differences in left SMG and ANG regions indicated a decreasing reliance on phonology‐based reading processes and an increasing reliance on visual mechanisms with increasing age, the authors emphasized that children and adults exhibited largely overlapping activation patterns during visual word processing.

The first quantitative coordinate‐based meta‐analysis of brain activation during reading in children (age means around 10 years) was conducted by Houdé et al. [2010] and identified similar left dorsal TP, ventral OT, and IFG circuits as a classical meta‐analysis on adult reading [Jobard et al., 2003]. The highly similar activation pattern in children and adults—in particular the marked left ventral OT and IFG activation—is surprising in the context of the proposed developmental shift of activation from left dorsal to ventral regions of the classical functional neuroanatomical model of reading, thus raising doubts on the validity of this model.

Further developmental functional magnetic resonance imaging (fMRI) and EEG studies indicated an early engagement of left ventral OT regions for print even before formal reading instruction, and correspondingly, an early failure of such an engagement in developmental dyslexia [Bach et al., 2010; Maurer et al., 2007; Raschle et al., 2012]. Importantly, training of letter‐speech sound correspondences in nonreading kindergarten children led to the emergence of sensitivity for print in the ventral OT cortex but not in the dorsal TP cortex, as would be expected from the classical dorsal/ventral functional neuroanatomical model of reading [Brem et al., 2010]. In a similar fashion, a recent meta‐analysis of age‐related dyslexic brain dysfunctions identified left TP underactivation (in relation to age‐matched nonimpaired readers) only in dyslexic adults but not in dyslexic children [Richlan et al., 2011]. Left OT underactivation, in contrast, was identified in both dyslexic children and dyslexic adults.

The present quantitative coordinate‐based meta‐analysis aimed to shed light on the engagement of brain regions in child and adult reading. We were specifically interested in the roles of the left dorsal TP cortex and of the left ventral OT cortex. We updated the set of studies included in the meta‐analysis by Houdé et al. [2010] and matched adult studies to the child studies to directly compare reading‐related activation in children to reading‐related activation in adults in a single meta‐analytic framework. Separate meta‐analyses were performed for the two age groups and directly compared in a meta‐analytic difference map. This strategy allowed for the identification and localization of brain regions exhibiting commonalities and differences between school‐aged children and experienced adult readers. This study is the first meta‐analysis comparing the reading systems of healthy children and adults without reading difficulties in an objective and quantitative way with the same meta‐analytic method.

For children, we expected to replicate the findings of Houdé et al. [2010], that is, activation in left OT, MTG, IPL, IFG, and SMA regions. For adults, we expected to find largely overlapping activation. Due to increasing functional specialization, however, we expected to identify an age‐related increase in activation in left OT and IFG regions (corresponding to predominance of reliance on stored visual–orthographic representations) and an age‐related decrease in activation in left TP regions (corresponding to abandonment of phonology‐based reading processes).

## MATERIALS AND METHODS

The selection of studies for the present coordinate‐based meta‐analysis was oriented on other meta‐analyses in the domain of reading [Houdé et al., 2010; Richlan et al., 2009, 2011]. Studies were selected when they met the following criteria: (1) healthy human participants were investigated with fMRI, (2) tasks were reading or reading‐related (e.g., rhyme judgments) with visual word, nonword, or letter string stimuli, (3) 3‐D coordinates of a single contrast against a low‐level baseline (fixation cross, rest, symbol, symbol strings, dots, or checkerboards) were reported in a standard stereotactic space (Talairach or MNI). Furthermore, to increase homogeneity, the selection was restricted to studies in alphabetic writing systems.

### Children

The recent meta‐analysis of Houdé et al. [2010] was used as a starting point for the selection of studies on reading in children (age means: 7–12 years). All studies selected by Houdé et al. [2010] were included, except for the study by Temple et al. [2001], who presented only single letters but not letter strings as specified by our selection criteria. Through several Medline/PubMed searches (http://www.pubmed.org) with the keywords “reading,”“language,”“brain imaging,” “fMRI,”“functional magnetic resonance imaging,” and “children” this selection was supplemented and updated. Furthermore, we checked the reference lists of each of the selected articles to identify additional relevant publications.

Based on the above criteria 19 studies were identified as suitable for inclusion in the meta‐analysis of the brain system for reading in children: Bach et al. [2010], Backes et al. [2002], Bitan et al. [2006, 2007a, 2007b], Blumenfeld et al. [2006], Booth et al. [2001, 2003, 2007a], Brem et al. [2009], Cao et al. [2006, 2008], Gaillard et al. [2001, 2003], Hoeft et al. [2006, 2007], Noble et al. [2006], Rimrodt et al. [2009], and Van der Mark et al. [2009]. Hoeft et al. [2006] used two control samples, one matched for age and one matched for reading ability for the comparison with dyslexic readers. Since activation was reported separately for each group, both control samples were included. In the following, we refer to the 20 included samples as 20 studies for reasons of simplicity.

Importantly, we decided to include all published studies of child reading that met our inclusion criteria, despite a potentially not perfect match with adult studies (see next section). The reasons for this are twofold. First, the inclusion of all child studies implies the usage of a wide range of different tasks. The meta‐analysis was expected to produce effects that are systematically found across studies, that is, effects that are independent of task. Second, a high number of included studies increase the power and reliability of the meta‐analyses.

### Adults

The search for adult studies was based on older meta‐analyses of the brain systems for language [Bolger et al., 2005; Ferstl et al., 2008; Jobard et al., 2003; Turkeltaub et al., 2002; Vigneau et al., 2006] and on the Medline/Pubmed searches described above with the keyword “adults” instead of “children.” To identify further relevant studies matching our inclusion criteria, the reference lists of each of the selected articles were checked. Twenty adult studies were matched to the 20 child studies in a pairwise manner based on the following criteria (in order of priority): (1) in‐scanner activation task and stimuli, (2) native language, and (3) sample size: Bitan et al. [2005], Booth et al. [2001, 2002, 2003], Brem et al. [2009], Binder et al. [2005, 2006], Burton et al. [2005], Chee et al. [1999], Cohen et al. [2002, 2003], Dehaene et al. [2001], Ferstl and von Cramon [2001], Kiehl et al. [1999], Mechelli et al. [2000], Poldrack et al. [2001], Rapp et al. [2004], Robertson et al. [2000], Tagamets et al. [2000], and Xu et al. [2005].

Some potentially relevant studies were not eligible for inclusion in the meta‐analysis because they only provided coordinates for the comparison between dyslexic and normal readers [Blau et al., 2010; Kronbichler et al., 2006; Meyler et al., 2008; Richlan et al., 2010; Schulz et al., 2008, 2009; Wimmer et al., 2010]. Furthermore, to clearly differentiate between children and adults, studies with adolescents were excluded [e.g., Grünling et al., 2004; Landi et al., 2010]. Several potentially interesting studies could not be included in the meta‐analysis because reading‐related activation was not reported separately for young and older readers [Brown et al., 2005; Schlaggar et al., 2002; Turkeltaub et al., 2003], or not in terms of 3‐D coordinates in standard stereotactic space on the whole‐brain level [e.g., Holland et al., 2001; Shaywitz et al., 2002, 2007], or not for an alphabetic language [Chinese: Cao et al., 2009; Chou et al., 2009; Tan et al., 2000; Siok et al., 2003; or Japanese: Nakamura et al., 2000, 2002; Miura et al., 2005]. This specific study selection was intended to improve the quality and accuracy of the meta‐analytic results. Studies requiring reading aloud were mostly excluded in favor of studies on silent visual word processing because additional requirements of overt articulation were not the focus of the present meta‐analysis. For another reason, reading aloud is more susceptible to cause motion artifacts in fMRI investigations, thereby leading to potentially lower data quality. As an exception, the study by Binder et al. [2005] was included in the set of adult studies albeit their participants were asked to read nonwords aloud. The study was one of the nonword reading studies that were included in the adult study set to balance the higher proportion of rhyming studies in the child study set (described in more detail below). The Binder et al. [2005] study is suitable for inclusion, as a response window was inserted between image acquisitions so that motion artifacts can be assumed to be minimized. Another reason to accept the exception was that activation reported for reading nonwords aloud was comparable to activation reported for reading nonwords silently.

A total number of 676 participants, 395 children (age means: 7–12 years) and 281 adults (age means: 23–34 years) were included in the 40 studies. Table 1 provides an overview of the selected studies and their main characteristics (for more details see Table SI, Supporting Information). The first column gives the indices of the study‐pairs that resulted from our matching procedure. The difference between child and adult studies with respect to total number of participants resulted from the fact that matching on sample size was allocated lower priority than matching on task/stimulus type and matching on language.

**Table 1:** Main characteristics of the included fMRI studies and number of foci used in the meta‐analysis

Pair	Year	First author	*N*	m	f	Native language	Age mean (SD; range)	Task type	Contrast	Voxel‐level (height) *p*<	Cluster‐level (extent) *p*< or no. of voxels	No. of foci	Design	Accuracy % correct mean (SD)	RT mean (SD) ms
*Children*															
1	2010	Bach	18	7	11	German	8.3 (0.4)	Word & nonwordmental letter substitution	W & NW > fixation	0.005 corr.	15 voxels	2	E‐r	83.3 (11.1)	2,787 (231)
2	2002	Backes	8	8	0	Dutch	11.6 (0.7; 11–12)	Nonword rhyme judgment	NW rhyming > fixation	0.0005 corr.	15 voxels	11	B	n/a	2,441 (207)
3	2006	Bitan	15	8	7	English	10.9 (9–12)	Word rhyme judgment	W spelling > symbols	0.001 unc.	35 voxels	7	B	89 (7)	1,529 (220)
4	2007a	Bitan	38	16	22	English	11.7 (9–15)	Word rhyme judgment	W spelling > symbols	0.001 unc.	10 voxels	7	E‐r	93 (1.7)	1,257 (65)
5	2007b	Bitan	36	14	22	English	(9–15)	Word rhyme judgment	W rhyming > fixation	0.0001 unc.	10 voxels	11	E‐r	86 (78–93)	1,384 (1,307‐1,461)
6	2006	Blumenfeld	16	8	8	English	10.7 (0.7; 9–12)	Semantic association judgment	W meaning > symbols	0.01 unc.	15 voxels	3	B	93.9 (3.7)	n/a
7	2001	Booth	5	5	0	English	11.1 (10–13)	Semantic association judgment	W meaning > symbols	0.001 unc.	12 voxels	5	B	89.9 (SE 2.7)	1,727 (SE 87)
8	2003	Booth	15	8	7	English	10.7 (9–12)	Semantic association judgment	W meaning > symbols	0.001 unc.	15 voxels	1	B	∼ 94	n/a
9	2007a	Booth	13	9	4	English	10.5 (2.19)	Semantic association judgment	W meaning > fixation	0.001 unc.	15 voxels	6	E‐r	96 (4)	1,276 (376)
10	2009	Brem	19	9	10	German	10.3 (9–12)	Word rhyme judgment	W reading > baseline	0.05 corr.	20 voxels	8	E‐r	98.19 (0.02)	613 (80)
11	2006	Cao	14	8	6	English	11.5 (9–14)	Word rhyme judgment	Conflicting trials > fixation	0.001 unc.	20 voxels	9	E‐r	79.5 (7.3)	1,240 (351)
12	2008	Cao	12	8	4	English	12.3 (9–14)	Word rhyme judgment	Lexical trials > fixation	0.001 unc.	10 voxels	9	E‐r	86 (10)	1,120 (265)
13	2001	Gaillard	9	4	5	English	10.2 (8–13)	Sentences reading	Reading fable > dots	0.01 corr.	0.05 corr.	6	B	n/a	n/a
14	2003	Gaillard	16	6	10	English	7.2 (6–8)	Sentences reading	Reading stories > dots	0.001 corr.	10 voxels	24	B	n/a	n/a
15	2006a	Hoeft	10	4	6	English	10.9 (0.3)	Word rhyme judgment	W rhyming > fixation (age‐matched)	0.01 unc.	10 voxels	11	B	90 (10.5)	2,445 (428)
16	2006b	Hoeft	10	5	5	English	8.7 (0.3)	Word rhyme judgment	W rhyming > fixation (reading‐level‐matched)	0.01 unc.	10 voxels	18	B	80 (12.5)	2,571 (431)
17	2007	Hoeft	64	27	37	English	10 (8–12)	Word rhyme judgment	W rhyming > fixation	0.01 corr.	10 voxels	9	B	78.4 (15.9)	n/a
18	2006	Noble	38	17	21	English	7.9 (7–10)	Letter string one‐back task	NW one‐back task > fixation	0.001 unc.	5 voxels	16	B	mean d‐prime 2.5 (0.93)	n/a
19	2009	Rimrodt	15	6	9	English	11.8 (1.4; 9–14)	Sentences reading(meaningfulness decision)	Sentence reading> word recognition	0.001 unc.	78 voxels	7	B	87.2 (9.2)	980 (255)
20	2009	Van der Mark	24	10	14	German	11.3 (0.4)	Phonological lexical decision	W reading > fixation	0.05 corr.	10 voxels	10	E‐r	94 (7)	1,033 (299)
*Adults*															
1	2000	Mechelli	6	5	1	English	24 (20–34)	Word & nonword reading	W & NW reading > fixation	0.05 corr.	5 voxels	11	B	n/a	n/a
2	2002	Cohen	7	1	6	French	(20–30)	Word & nonword reading	W & NW reading> checkerboard	0.01 unc.	0.05 corr.	8	B	77.85 (outside scanner)	n/a
3	2005	Bitan	14	5	9	English	(20–35)	Word rhyme judgment	W spelling > symbols	0.001 unc.	50 voxels	6	B	98 (2)	868 (196)
4	2002	Booth	13	3	10	English	24.6 (20–35)	Word rhyme judgment	W spelling > symbols	0.001 unc.	11 voxels	7	B	96.8 (SE 5.1)	899 (SE 93)
5	2005	Binder	24	12	12	English	27.5 (18–48)	Nonword reading	NW reading > fixation	0.001 unc	0.05 corr.	97	E‐r	96.8 (3.3)	966 (158)
6	1999	Kiehl	6	6	0	English	24.5 (22–36)	Word & nonword reading(lexical decision)	W & NW reading > symbols	0.05 ‐ 0.001 corr.	n/a	19	B	n/a	627 (90)
7	2001	Booth	4	4	0	English	25.5 (22–31)	Semantic association judgment	W meaning > symbols	0.001 unc.	12 voxels	5	B	90.9 (SE 3.3)	1,666 (SE 126)
8	2003	Booth	15	7	8	English	28.8 (21–36)	Semantic association judgment	W meaning > symbols	0.001 unc.	15 voxels	2	B	∼ 98	n/a
9	1999	Chee	8	5	3	English	(22–38)	Word reading(abstract/concrete decision)	W meaning > fixation	0.00001 unc.	n/a	29	B	92.6	850
10	2009	Brem	18	9	9	German	25.2 (20–31)	Word rhyme judgment	W reading > baseline	0.05 corr.	20 voxels	9	E‐r	99.31 (0.01)	519 (70)
11	2005	Burton	14	6	8	English	26.7 (6; 21–46)	Word & nonword rhyme judgment	W, NW, SQ > single FF	0.005 unc.	8 voxels	10	B	99 (SE 0.01)	1,483 (46)
12	2003	Cohen	9	2	7	French	60 (42–68)	Word & nonword reading	W & NW reading > fixation	0.005 unc	0.05 corr.	11	B	100	539
13	2000	Robertson	8	4	4	English	n/a	Sentences reading	Sentence reading > symbol strings	0.001 unc.	0.05 corr.	8	B	83	n/a
14	2001	Ferstl	12	8	4	German	23 (3.1; 19–31)	Sentences reading (coherence decision)	Sentence reading >letter case judgment	0.001 unc.	> 125 mm3	10	E‐r	94 (3)	2,072 (542)
15	2001	Dehaene	37	12	25	French	(19–34)	Word readingmasked & unmasked	Visible words > fixation	0.001 unc.	0.05 corr.	15	E‐r	88.9 (outside scanner)	n/a
16	2006	Binder	30	15	15	English	(18–49)	Nonword reading (contained an ascender letter)	Letter string task > dots	0.00001 unc.	0.05 corr.	31	E‐r	99.1 (1.5)	726 (100)
17	2001	Poldrack	8	4	4	English	(20–29)	Nonword rhyme judgment	Rhyme judgment >letter case judgment	0.05 corr.	10 voxels	2	B	88	n/a
18	2000	Tagaments	11	6	5	English	28 (20–47)	Word one‐back task	W one‐back task > symbols	0.05 corr.	n/a	18	B	n/a	n/a
19	2005	Xu	22	22	0	English	34 (12; 21–65)	Sentence reading	Sentence reading > letter strings	0.001 unc.	n/a	17	B	100 (outside scanner)	n/a
20	2004	Rapp	15	9	6	German	(19–51)	Sentence reading	Literal sentences >low level baseline	0.05 corr.	10 voxels	12	E‐r	94.2 (5.8)	2,140 (580)

Thirty of the included studies were conducted with English participants, six with German participants, three with French participants, and one with Dutch participants. Thirteen child–adult study pairs investigated English and two child–adult study pairs investigated German participants. Further, one child study investigated German and the matched adult study investigated English participants. For another child–adult study pair, it was vice versa. In addition, two child studies that used English participants were matched to adult studies that used French participants. We classified French as deep orthography, referring to Paulesu et al. [2000], who conducted a seminal cross‐language brain imaging study on reading. The remaining child–adult study pair used Dutch and French participants. In sum, the meta‐analytic set of child studies included 16 studies in deep orthographies and four studies in shallow orthographies, whereas the meta‐analytic set of adult studies included 17 studies in deep orthographies and three studies in shallow orthographies.

To ensure statistical independence of the coordinates extracted from each study, only local maxima from a single activation condition contrasted against a low‐level baseline (fixation cross, rest, symbol, symbol strings, dots, or checkerboards) was included per study. For the child studies, the reading‐related activation condition resulting in the highest number of activation foci was always selected. Accordingly, for the adult studies the condition most closely matching the activation condition of the child studies was selected. Five child–adult study pairs used word/nonword rhyme judgments, four pairs used semantic decisions, three pairs used sentence reading, one pair used word/nonword reading, and one pair used one‐back tasks. For the remaining six child studies, no directly matching adult study with respect to our primary matching criterion (in‐scanner activation task) could be identified: five child studies used word/nonword rhyme judgments and one used phonological lexical decisions. Therefore, the remaining six child‐adult study pairs were matched with respect to the following study characteristics: native language (three pairs), orthographic depth of the language (two pairs), sample size (two pairs), control condition (three pairs), and approximate number of foci (three pairs). Of the six adult studies, five used word/nonword reading and one used sentence reading. In sum, each meta‐analytic set included five phonological, four semantic, three sentence reading tasks, one word/nonword reading task, and one one‐back task. While the set of adult studies included one additional sentence reading and five word/nonword reading tasks, the set of child studies included six additional phonological tasks. To account for this higher proportion of phonological tasks in the set of child studies, the set of adult studies included a higher proportion of studies that used nonword stimuli, which require phonological processing (three child studies vs. eight adult studies).

All of the 40 studies reported foci of reading‐related brain activation, resulting in a total number of 507 foci (180 for child reading and 327 for adult reading) entering the meta‐analysis. The imbalance between child and adult studies with respect to the total number of foci does not affect the direct statistical comparison, since the meta‐analytic method (see below) compares the mean maps of the two sets of studies [Radua and Mataix‐Cols, 2009]. Nevertheless, we will comment on possible limitations of comparing different age groups in the Discussion section.

Another issue is related to the behavioral performance during the in‐scanner activation tasks. On average, children exhibited lower accuracy (*M*_Children_ = 88.65% vs. *M*_Adults_ = 93.91%, *t*(31) = −2.41, *P* = 0.01) and longer reaction times (*M*_Children_ = 1,600 ms vs. *M*_Adults_ = 1,113 ms, *t*(24) = 1.94, *P* = 0.03) compared with adults. This finding reflects the natural situation of higher reading‐related skills in adults and certainly has to be taken into account when interpreting age‐related group differences in brain activation (see Discussion section). From the reasonable accuracy of the children (at least around 80% correct), however, we can be certain that the tasks were still manageable for the young readers and that they did not exhibit a complete overload/breakdown of the reading system.

An additional issue is related to the brain template used for spatial normalization in the child studies. The Supporting Information (S1 right most column) shows that normalization in child studies was handled similarly to that in the adult studies, that is, the standard T1 MNI template was used. This procedure seems justified as neuroanatomical differences between children and adults are unlikely to affect fMRI results [Booth et al., 2001; Brem et al., 2009; Hoeft et al., 2006, 2007]. Hoeft et al. [2007] used both, the standard adult template as well as an adjusted template including all their child participants (between the ages of 8.2 and 12.4 years) and found similar results in terms of location and statistical significance.

For the present coordinate‐based meta‐analysis, Anisotropic Effect‐Size Signed Differential Mapping (ES‐SDM) software (http://www.sdmproject.com), version 4.13 was used [Radua and Mataix‐Cols, 2009; Radua et al., 2010, 2012, 2014]. The idea behind ES‐SDM is to recreate a statistical parametric map of effect sizes (Hedge's *g*) for each original study based on the reported activation foci, their respective statistical values, and the number of included participants. The special feature of Anisotropic ES‐SDM compared with traditional ES‐SDM is that it accounts for spatial anisotropy of activation clusters due to anatomical constraints.

All foci reported by the original studies were transformed to MNI space with a built‐in feature using the icbm2tal transform [Lancaster et al., 2007]. Meta‐analysis was restricted to a specific gray matter template provided by the software. For each study, effect size maps (Hedge's *g*) were recreated by convolving reported activation foci with a fully anisotropic unnormalized Gaussian kernel (*α* = 1). The anisotropy of the kernel was based on the spatial correlations of the gray matter template. Within a study, values obtained by close anisotropic kernels were combined by square‐distance‐weighted averaging. A random effects general linear model was used to combine the data across study‐specific effect size maps. To examine statistical significance, the location of activation foci was permuted within the gray matter template (500 randomizations). Finally, the meta‐analytic maps were thresholded using a voxel‐level (height) threshold of *P* < 0.005 (uncorrected), and a cluster‐level (extent) threshold of 10 voxels (corresponding to the default settings of Anisotropic ES‐SDM). For ES‐SDM, this uncorrected threshold was found to optimally balance sensitivity and specificity, and to be an approximate equivalent to a corrected threshold of *P* < 0.05 in original neuroimaging studies [Radua et al., 2012].

We computed two separate meta‐analytic maps for children and adults to investigate regions of consistent activation across studies in each age‐specific set. In addition, we computed a difference map by subtracting the ES‐SDM values of the map for children from the ES‐SDM values of the map for adults. This difference map was thresholded at the same voxel‐level and cluster‐level thresholds used for the separate maps. It provides a direct statistical comparison of child and adult reading activation, thus informing on reliable age‐related differences.

To evaluate the robustness of the meta‐analytic findings, we used systematic whole‐brain voxel‐based jackknife sensitivity analysis (implemented in the SDM software). This procedure consists of repeating the separate meta‐analyses for children and adults for the number of included studies and each time excluding a different study (i.e., the separate meta‐analyses were repeated 20 times, with a different combination of 19 included studies each). The idea is that if a meta‐analytic finding remains statistically significant in all or most of the combinations of studies, it can be concluded that this finding is robust against changes of the sample, and thus, is highly replicable [Radua and Mataix‐Cols, 2009]. Furthermore, we inspected how many of the original studies contributed to the identification of each meta‐analytic cluster. This method was already applied in previous meta‐analyses from our lab [Richlan et al. 2009, 2011, 2013] and provides a straightforward assessment of the consistency of meta‐analytic findings.

## RESULTS

**Figure 1:**
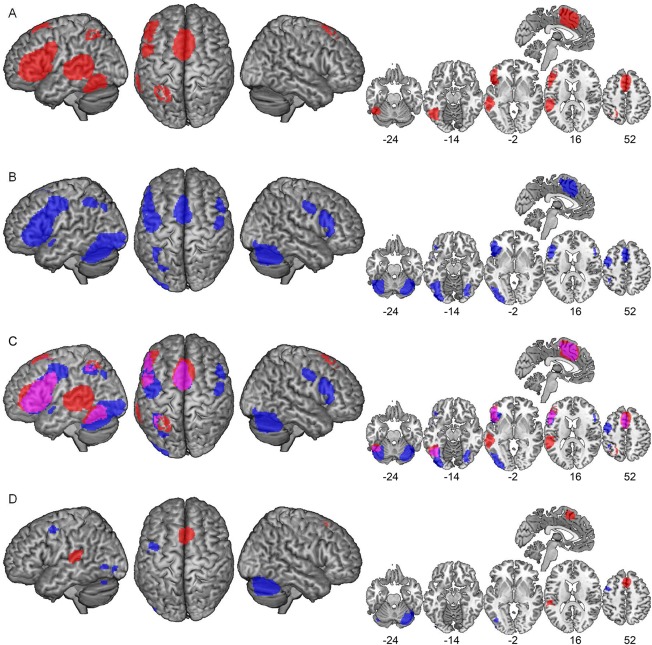
(**A**) Surface rendering and selected slices of the separate meta‐analytic map of reading‐related activation in children (red). (**B**) Surface rendering and selected slices of the separate meta‐analytic map of reading‐related activation in adults (blue). (**C**) Surface rendering and selected slices of both separate meta‐analytic maps. Overlapping regions are shown in violet. (**D**) Surface rendering and selected slices of the meta‐analytic difference map for the direct comparison between children (red) and adults (blue).

The reading system of children identified in the present meta‐analysis is shown in Figure 1A (rendered on a template brain) and Table 2. Clusters identified with convergent reading‐related activation in children are characterized by the MNI coordinates, the SDM‐*Z* values of local maxima, and the extent of the clusters. Specifically, the meta‐analysis identified a large bilateral cluster around the SMA. Another large cluster was localized in the left IFG including opercular and triangular parts and extending into middle frontal (MFG) and PRG. Furthermore, the meta‐analysis identified a large cluster in the left OT cortex with maxima in ITG, MTG, and STG and extending into FFG and inferior occipital gyri (IOG). With respect to spatial extent, the bilateral SMA cluster was the largest, followed by the left IFG and the left OT clusters. Additionally, one smaller cluster was localized in the left posterior parietal cortex (PPC).

**Table 2:** Results of the separate meta‐analysis of reading‐related activation in children

	MNI coordinates					
Region	*x*	*y*	*z*	SDM‐*Z*	Voxels	JK
Bilateral supplementary motor area					3,426	20
L supplementary motor area, BA 6	−2	24	56	8.547		
R supplementary motor area, BA 6	4	8	50	7.911		
L inferior frontal cortex					2,809	20
L inferior frontal gyrus, BA 44	−50	22	4	6.343		
L inferior frontal gyrus, BA 45	−52	24	18	5.170		
L middle frontal gyrus	−48	26	24	4.949		
L precentral gyrus, BA 6	−56	4	32	4.326		
L occipito‐temporal cortex					3,433	20
L inferior temporal gyrus	−52	−60	−14	5.175		
L superior temporal gyrus	−56	−32	16	5.116		
L middle temporal gyrus	−58	−26	−6	4.306		
L posterior parietal cortex					128	20
L superior parietal lobule, BA 7	−22	−52	52	3.880		

*Note*. BA = Brodmann area, L= left; R= right; JK = jackknife analysis (number of subsamples that replicate the finding).

The reading system of adults is shown in Figure 1B and Table 3. For convergent reading‐related activation in adults, the meta‐analysis identified a large left IFG cluster including opercular and triangular parts and further maxima in PRG and MFG. Another large left hemisphere cluster was localized in the left OT cortex with maxima in FFG, IOG, ITG, and middle occipital gyrus (MOG). Moreover, a large bilateral SMA cluster was identified. Additionally, two smaller clusters were located in the left PPC and in the left temporal pole. In the right hemisphere there was a large cerebellar cluster reaching into the right ventral OT cortex. Further smaller right hemisphere clusters were identified in IFG (including opercular and triangular parts) and in the PRG. With respect to spatial extent, the left IFG cluster was the largest followed by the left OT, the right cerebellar, and the bilateral SMA clusters.

**Table 3:** Results of the separate meta‐analysis of reading‐related activation in adults

	MNI coordinates					
Region	*x*	*y*	*z*	SDM‐*Z*	Voxels	JK
L inferior frontal cortex					3,856	20
L inferior frontal gyrus, BA 45	−52	20	18	5.889		
L inferior frontal gyrus, BA 44	−52	18	14	5.774		
L precentral gyrus, BA 6	−46	2	42	5.211		
L middle frontal gyrus	−42	4	48	5.111		
R cerebellum					2,622	20
R cerebellum	32	−70	−28	5.864		
L occipito‐temporal cortex					3,412	20
L fusiform gyrus	−42	−68	−22	5.306		
L inferior occipital gyrus	−44	−74	−4	5.204		
L middle occipital gyrus	−42	−86	−2	4.894		
L inferior temporal gyrus	−48	−62	−20	4.752		
Bilateral supplementary motor area					1,853	20
L supplementary motor area, BA 6	−4	24	56	4.652		
R supplementary motor area, BA 6	2	6	50	4.585		
R inferior frontal cortex					389	20
R inferior frontal gyrus, BA 45	52	20	20	4.239		
R inferior frontal gyrus, BA44	52	16	32	3.970		
L posterior parietal cortex					262	20
L intra‐parietal sulcus	−42	−48	48	4.124		
R precentral gyrus					187	20
R precentral gyrus, BA 4	52	−8	42	3.711		
L temporal pole					16	18
L temporal pole	−52	4	−10	3.427		

Note. BA = Brodmann area, L= left; R= right; JK = jackknife analysis (number of subsamples that replicate the finding).

For further evaluation of convergence, Tables 4 and V show which of the original studies reported one or more activation foci contributing to the identified meta‐analytic clusters. Furthermore, additional findings of the original studies, which found no support in the present meta‐analysis, are reported in the rightmost column of Tables 4 and V, respectively. Due to little consistency across studies these regions did not result in statistically significant meta‐analytic clusters.

**Table 4:** Convergence across child studies

Year	First author	Bilateral supplementary motor area	L inferior frontal cortex	L occipito‐temporal cortex	L posterior parietal cortex	Additional regions
2010	Bach	X	X			
2002	Backes	X	X	X	X	
2006	Bitan	X	X	X	X	L lentiform nucleus, R FFG,
2007a	Bitan	X	X	X	X	L thalamus, calcarine sulcus
2007b	Bitan	X	X	X	X	L putamen, thalamus, R IFG, FFG
2006	Blumenfeld			X		R MFG, ITG
2001	Booth		X	X		R cerebellum
2003	Booth			X		
2007a	Booth	X	X	X		R MFG, FFG, thalamus
2009	Brem	X	X	X		R STG, R MTG,L lentiform nucleus, R IOG
2006	Cao	X	X	X	X	R FFG, thalamus
2008	Cao	X	X	X		R FFG
2001	Gaillard	X	X	X		R MTG, LIG
2003	Gaillard	X	X	X		L thalamus, B precuneus, R STG, MTG, IOG, cerebellum
2006a	Hoeft	X	X		X	B IFG, SPL MOG, cerebellum, R STG
2006b	Hoeft	X	X	X		L lentiform nucleus, thalamus, R FFG, MOG, cerebellum, claustrum
2007	Hoeft	X	X	X	X	R IPL, SPL
2006	Noble	X	X	X		L medial cerebellum, R IOG, MOG, ITG, ANG, PRG
2009	Rimrodt	X	X			R FFG, cerebellum, LIG, cuneus
2009	Van der Mark	X	X	X		R STG, PRG, insula
	Total	17	18	17	7	

Studies reporting activation foci in the regions presently identified with reading‐related activation in children are marked with an X. Furthermore, findings of activation in additional brain regions are reported.

*Note*. ANG= angular gyrus; B= bilateral; FFG= fusiform gyrus; IFG= inferior frontal gyrus; IOG= inferior occipital gyrus; IPL= inferior parietal lobule; ITG= inferior temporal gyrus; L= left; LIG= lingual gyrus; MFG= middle frontal gyrus; MOG= middle occipital gyrus; MTG= middle temporal gyrus; PRG= precentral gyrus; R= right; SPL= superior parietal lobule.; STG= superior temporal gyrus.

**Table 5:** Convergence across adult studies

Year	First author	L inferior frontal cortex	R cerebellum	L occipito ‐temporal cortex	Bilateral supplementary motor area	R inferior frontal cortex /precentral gyrus	L posterior parietal cortex	L temporal pole	Additional regions
2000	Mechelli		X	X			X		R SPL
2002	Cohen	X		X	X	X			R IPL
2005	Bitan	X		X	X	X	X		R SPL
2002	Booth	X	X	X	X	X			
2005	Binder	X	X	X	X	X	X		R IPS, L midbrain, subcortical (8 foci)
2006	Kiehl	X	X	X	X	X	X		R IPL, STG
2001	Booth	X	X	X					Cuneus
2003	Booth		X	X					
2007	Chee	X	X	X	X	X	X		Basal ganglia
2009	Brem	X	X	X	X			X	L caudate, lentiform nucleus
2005	Burton	X	X	X					L thalamus
2003	Cohen	X	X	X		X	X		L thalamus, B lentiform nucleus
2001	Ferstl	X	X		X	X		X	R STG, precuneus
2000	Robertson	X		X					L parahippocampal gyrus R MTG
2001	Dehaene	X	X	X	X	X	X		R IPS
2006	Binder	X	X	X	X	X	X		R IPS, thalamus, putamen
2001	Poldrack	X							
2000	Tagamets	X	X	X	X			X	
2005	Xu	X	X	X	X	X		X	L putamen, L thalamus
2004	Rapp	X		X	X	X	X	X	L thalamus, R lentiform nucleus
	Total	18	15	18	13	12	9	5	

Studies reporting activation foci in the regions presently identified with reading‐related activation in adults are marked with an X. Furthermore, findings of activation in additional brain regions are reported.

*Note*. B= bilateral; IPL= inferior parietal lobule; IPS= intra‐parietal sulcus; L= left; MTG= middle temporal gyrus; R= right; SPL= superior parietal lobule.; STG= superior temporal gyrus.

For children, highest convergence across studies was found for the left IFG cluster with 18 studies (out of 20) contributing. In addition, substantial convergence was found for the bilateral SMA cluster and for the left OT cluster with 17 studies contributing each. To increase regional specificity and to facilitate comparison with the adult meta‐analysis, the left OT cluster was divided into a ventral part (including ITG) and a dorsal part (including MTG and STG). Sixteen studies contributed to the ventral part and 11 studies contributed to the dorsal part. The remaining meta‐analytic cluster in the left PPC showed limited convergence across studies with seven studies contributing.

For adults, highest convergence across studies was found for the left IFG and the left ventral OT clusters with 18 studies (out of 20) contributing each. Fifteen studies contributed to the right cerebellar cluster, 13 studies contributed to the bilateral SMA cluster, and 12 studies contributed to the right frontal clusters. Similar to the results of the meta‐analysis for children, the left PPC cluster and the left temporal pole cluster showed limited convergence with nine and five studies contributing, respectively.

The left ventral OT and left IFG clusters showed similarly high convergence across the child and adult studies. In these regions, children and adults showed substantial overlap of activation and the high number of studies contributing to the identification of the meta‐analytic clusters speaks for high consistency within each age‐specific set of studies and robustness of the meta‐analytic results. Convergence for the left dorsal OT and bilateral SMA clusters was more pronounced for the child studies, whereas convergence for right hemisphere clusters was more pronounced for the adult studies.

For evaluation of the replicability, we used jackknife sensitivity analysis. Tables 3 and IV show that the meta‐analytic findings remained unchanged in most recalculations of the meta‐analysis, indicating robustness against changes of the sample. For the meta‐analysis of child studies, we found perfect replicability of findings in bilateral SMA and in left inferior frontal, occipito‐temporal, and PPC (20 out of 20 leave‐one‐out recalculations). Similarly, for the meta‐analysis of adult studies findings were perfectly replicable (20 out of 20 leave‐one‐out recalculations, respectively), with one exception: left temporal pole activation was identified by 18 out of 20 leave‐one‐out recalculations.

To identify overlapping regions in children and adults (marked in purple), Figure 1C shows the separate meta‐analytic maps for children and adults rendered on a template brain. An overlapping region was located in the left ventral OT cortex, more specifically in FFG, ITG, and cerebellar regions (*y* = −44 to −75). With respect to this left ventral OT cluster, convergent activation in children (Figure 1C, marked in red) extended more laterally and dorsally into superior and middle temporal regions (*y* = −12 to −47). In contrast, convergent activation in adults (Figure 1C, marked in blue) extended into medial, posterior, and inferior directions (*y* = −76 to −102). Furthermore, a substantial left IFG cluster (*y* = 49 to −4) was part of the reading system of both children and adults. For both age groups, this cluster included opercular and triangular parts and reached into the MFG, PRG, and insula. For the adult readers, the left frontal cluster extended into posterior (between *y* = −5 and *y* = −19) and superior (*z* = 46–58) areas that were not identified in children. In contrast, the bilateral SMA cluster was identified for both age groups (*y* = 36 to −8), but was more extended overall in child readers. Moreover, an overlapping region was identified for both age groups in the left PPC. Convergent activation in children extended medially (*x* = −22 to −18) and dorsally (*z* = 56–62) into superior parietal regions. In contrast, convergent activation in adults extended posterior (*y* = −68 to −76) and inferior (*z* = 34–42) into the ventral half of the intraparietal sulcus.

To obtain statistically reliable information on age‐related effects, we directly compared the children and adult studies in a meta‐analytic difference map. This direct statistical comparison identified two clusters with higher convergence in child readers compared with adult readers (Table 6 and Figure 1D, marked in red): one in the bilateral SMA (*x* = 2, *y* = 24, *z* = 54) and one in the left posterior STG (*x* = −56, *y* = −32, *z* = 16). In contrast, higher convergence in adult readers compared with child readers (Table 6 and Figure 1D, marked in blue) was identified in four left and one right hemisphere cluster. Small left hemisphere clusters were localized in the frontal cortex (MFG/PRG) (*x* = −46, *y* = 6, *z* = 52), in the MOG (*x* = −38, *y* = −72, *z* = −2 and *x* = −42, *y* = −88, *z* = −14) and in the cerebellum (*x* = −40, *y* = −70, *z* = −24). The large right hemisphere cluster was localized in the cerebellum (*x* = 34, *y* = −72, *z* = −28) and extended into the ventral OT cortex.

**Table 6:** Results of the meta‐analytic difference map for the direct comparison between children and adults

	MNI coordinates				
Region	*x*	*y*	*z*	SDM‐*Z*	Voxels
*Children > adults*					
Bilateral supplementary motor area					731
R supplementary motor area, BA 6	2	24	54	−2.747	
L superior temporal gyrus					313
L superior temporal gyrus	−56	−32	16	−2.060	
*Adults > children*					
R cerebellum					2147
R cerebellum	34	−72	−28	3.897	
L frontal cortex					89
L middle frontal gyrus	−46	6	52	2.743	
L precentral gyrus, BA 6	−52	−4	52	2.275	
L middle occipital gyrus					55
L middle occipital gyrus	−38	−72	−2	2.742	
L middle occipital gyrus					31
L middle occipital gyrus	−42	−88	−14	2.732	
L cerebellum					21
L cerebellum	−40	−70	−24	2.456	

*Note*. BA = Brodmann area, L= left; R= right.

## DISCUSSION

The goal of the present meta‐analysis was to identify age‐related commonalities and differences in brain activation during reading in children (age means: 7–12 years) and adults (age means: 23–34 years). We found activation common to the two separate meta‐analyses of children and adults in a widespread core network of left ventral OT, left IFG, left PPC, and bilateral SMA regions. Differences in the direct statistical comparison between children and adults were evident with respect to both extent and anatomical location of activation clusters. Specifically, higher meta‐analytic convergence of activation in child studies was identified in the bilateral SMA and in the left STG. In contrast, higher meta‐analytic convergence of activation in adult studies was identified in the bilateral cerebellum and in left MFG/PRG and MOG regions. Noteworthy, the identified differences between the two meta‐analytic maps of children and adults do not mean that reading‐related activation is higher in one age group than in the other. Rather, it means that the observed activation is more consistent across studies in one group than in the other.

The results of the separate meta‐analysis for children are largely in line with the recent meta‐analysis by Houdé et al. [2010], which included a smaller number of original studies and identified convergent activation for child reading in left dorsal TP, ventral OT, SMA, and IFG regions. Furthermore, the results of the separate meta‐analysis for adults resemble previous narrative reviews and systematic meta‐analyses emphasizing the important roles of ventral OT and IFG regions in skilled reading [Ferstl et al., 2008; Fiez and Petersen, 1998; Jobard et al., 2003; Taylor et al., 2013; Vigneau et al., 2006]. In the following sections, we will relate our findings to evidence from the literature and discuss potential implications on functional neuroanatomical models of reading.

### Ventral OT cortex

In the ventral OT cortex, we identified convergent reading‐related activation in child studies in a left anterior region (ITG) and activation in adult studies in extended bilateral regions including ITG, FFG, IOG, and MOG. The finding of specific ventral OT activation in children is in line with the notion of an early role of this region in learning to read [Brem et al., 2009; Church et al., 2008; Gaillard et al., 2003; Turkeltaub et al., 2003]. In addition, specific left ventral OT activation for visual words was reported even in prereading children after an 8‐week training of grapheme–phoneme correspondences [Brem et al., 2010]. With respect to developmental dyslexia, several studies found functional and structural left ventral OT abnormalities before learning to read in children later diagnosed as dyslexic [Bach et al., 2010; Maurer et al., 2007] or with a high familial risk for dyslexia [Raschle et al., 2012]. Taken together, these findings speak for an early engagement of left ventral OT regions in normal reading development and an early failure of such engagement in dyslexia.

Anatomically, the region where both children and adults exhibited reading‐related activation in the present meta‐analysis was located in anterior and middle parts of the left ventral OT cortex ranging from *y* = −44 to −76. This segment contains the VWFA [Cohen et al., 2000, 2002; McCandliss and Noble, 2003] or Visual Word Form System [Maurer et al., 2011; Van der Mark et al., 2009]. The local maximum of the child ventral OT activation (*x* = −52, *y* = −60, *z* = −14) was very close to the classical VWFA location (*x* = −45, *y* = −57, *z* = −12) with an Euclidean distance of only 8 mm. Likewise, the local maximum of the adult ventral OT activation (*x* = −48, *y* = −62, *z* = −20) was located only 10 mm away from the classical VWFA location. Thus, it is evident that both the child and adult ventral OT clusters covered the same parts of the left ventral visual pathway and that these clusters included the VWFA.

The VWFA was originally proposed to host prelexical visual orthographic representations based on orthographic regularities that constrain letter combinations in a given orthography [Cohen and Dehaene, 2004; Dehaene and Cohen, 2011; Dehaene et al., 2002, 2005; Vinckier et al., 2007]. From the beginning, the existence of a cortical region specialized for the processing of visual word forms was a matter of significant debate [Price and Devlin, 2003, 2004]. An alternative account posited that the left ventral OT cortex acts as an interface area between sensory bottom‐up input and higher‐level top‐down representations [Price and Devlin, 2011], but does not host stored orthographic representations. Other accounts went even further than the original VWFA account and proposed the existence of stored lexical visual orthographic entries in the VWFA (i.e., a mental lexicon) [Glezer et al., 2009; Kronbichler et al., 2004, 2007, 2009; Ludersdorfer et al., 2013; Schurz et al., 2014a]. Recently, it was found that left ventral OT activation was insensitive to the length of words but increased with increasing number of letters in pronounceable nonwords [Richlan et al., 2010; Schurz et al., 2010]. This length by lexicality interaction was interpreted as reflecting a double function of the left ventral OT cortex in both visual whole‐word recognition and phonological decoding (i.e., grapheme‐phoneme conversion). This account also explains the early engagement of the left ventral OT cortex in beginning readers and it's tuning to print after training of grapheme‐phoneme correspondences in kindergarten [Brem et al., 2010].

The results from our direct statistical comparison between child and adult studies identified higher convergence in adult studies in bilateral posterior ventral OT and cerebellar regions. Based on the classical neuroanatomical model of visual word recognition [e.g., Pugh et al., 2000] one would have predicted higher convergence in adult studies in the left anterior ventral OT including the VWFA [see also Shaywitz et al., 2002]. Interestingly, the overlap of the separate meta‐analyses covered the VWFA whereas the difference was identified in posterior ventral OT regions.

This finding is of further interest with respect to the Local Combination Detectors (LCD) model of Dehaene et al. [2005]. This model is built on a hierarchy of neurons sensitive to increasingly larger fragments of words along the posterior‐to‐anterior direction of the ventral visual pathway, from oriented bars, letter contours, letter shapes, abstract letter identities, and local bigrams to small words and recurring substrings. The visual clusters identified with higher convergence in adult compared with child studies correspond to the stages of letter shapes and letter contours. With respect to the LCD model one would have expected similar or even higher convergence in the child studies. The corresponding assumption is that children use the posterior portion of the system more than adults do because whole‐word recognition is still not automatized in children.

The developmental increase in bilateral posterior ventral OT activation is also of interest in relation to the Interactive Account of Price and Devlin [2011]. This model explains ventral OT activation according to the predictive coding framework; that is, according to the interaction of forward and backward connections. Specifically, it is assumed that backward connections predict the response of the forward connections and that the difference between these two (i.e., the prediction error) is minimized through recurrent neuronal message passing. Following this theorizing, we speculate that the higher convergence in adult compared with child studies in posterior ventral OT regions indicates increased backward connectivity from anterior to posterior stages of the ventral visual pathway as a consequence of increased reading expertise. Based on the Reverse Hierarchy Theory of visual perceptual learning by Ahissar and Hochstein [2004], it is plausible that learning to read is a top‐down guided process, which first leads to changes in high‐level anterior regions of the ventral OT cortex and only later progresses backward to low‐level posterior ventral OT regions. This tuning process is likely to operate through a decrease of activation in response to irrelevant stimuli. This means that the fact that posterior ventral OT regions were not identified in the meta‐analysis of child studies was probably due to similarly high activation for letter strings and control strings (e.g., symbols) in the original studies. In other words, the posterior ventral OT regions were active in children, but to a similar degree for both relevant (experimental) and irrelevant (control) stimuli, resulting in absence of reported reading‐related activation. Interestingly, a recent meta‐analysis of age‐related dysfunctions in dyslexia identified underactivation in dyslexic adults but not in dyslexic children in a very similar left posterior ventral OT region [Richlan et al., 2011].

Finally, Paulesu et al. [2000] who investigated cultural differences in reading reported higher STG activation in Italian compared with English readers and higher vOT activation in English compared with Italian readers. While readers of a shallow orthography like Italian can rely on phonology‐based reading processes (i.e., grapheme–phoneme conversion), readers of a deep orthography like English have to rely more on whole‐word recognition due to the often ambiguous grapheme–phoneme mapping. The present meta‐analytic findings show some similarities to the findings of Paulesu et al. [2000] and thus may be explained by properties of the writing systems of the included studies rather than by a change in reading strategies. Given that the proportion of deep and shallow orthographies in the included studies is balanced between the child and the adult study set, our findings speak for a change in reading strategies.

### Cerebellum

The cerebellum was identified with convergent reading‐related activation in adults but not in children. This finding received further support in the meta‐analytic difference map, where particularly the right cerebellum was shown to exhibit significantly higher convergence in adult compared with child studies. A meta‐analysis of the functional topography in the human cerebellum identified activation in right cerebellar regions, including lobules VI and crus I/II for language processing [Stoodley and Schmahmann, 2009]. In addition to evidence suggesting a functional role of the cerebellum in language processing [e.g., Keren‐Happuch et al., 2014; Marien et al., 2001; Stoodley, 2012; Stoodley et al., 2012] emerging evidence indicated the involvement in cognitive processes that are integral to reading [e.g., Fulbright et al., 1999; Turkeltaub et al., 2002, 2003]. More recently, the cerebellum was suggested to be an important part of the neural network supporting reading in typically developing readers [Stoodley and Stein, 2013; Travis et al., in press]. Booth et al. [2007b] provided evidence for reciprocal functional connections between the cerebellum and left inferior frontal and left lateral temporal regions. With respect to the present findings, these connections may not be fully developed in children and thus the cerebellum was identified only in the adult studies.

Lesions of the cerebellum have been associated with reading difficulties [Stoodley and Stein, 2013] and functional and anatomical abnormalities in this part of the brain have been associated with developmental dyslexia [e.g., Nicolson and Fawcett, 2011]. Nicolson and Fawcett [2005] described the core deficits of dyslexia in terms of poor skill automaticity. As skill automaticity was linked to cerebellar functions, the present finding may reflect that reading in children is not as automatized as in adults.

With respect to this difference between children and adults, one might speculate that the finding may be caused by differences in the size of the field of view (FOV) used for functional data acquisition in the original studies. To check whether the FOV covered the cerebellum to a greater extent in the adult compared with the child studies, we extracted the extension of the FOV in the *z*‐direction, that is, from superior to inferior, from each original study (Table 1 rightmost column). Considering the mean extension of both groups (*M*_adults_ = 114.4 mm; *SD* = 32.5 vs. *M*_children_ = 115.2 mm; *SD* = 16.5) and the fact that the lowest FOV extension was found in an adult study, we can conclude that the cerebellum was not covered better in adult than in child studies.

It is less clear to what extent the border between vOT and cerebellar activation was influenced by smoothing and normalization of the original studies. Spatial smoothing of fMRI brain activation data may artificially merge activation from adjacent albeit functionally and anatomically distinct brain regions [e.g., Fransson et al., 2002; Geissler et al., 2005; White et al., 2001]. Thus, the meta‐analytic finding of activation in the cerebellum may be influenced by smoothing or normalization of the original studies or may be a result of smoothing in the meta‐analysis. Given that, however, nine out of the 18 adult studies that contributed to the left vOT/cerebellar cluster reported distinct activation clusters in both, vOT and cerebellum, the results speak for activation in both regions.

### Inferior Frontal and Precentral Cortex

In the left IFG, we identified convergent reading‐related activation in both children and adults. In both groups, the cluster was localized in the left IFG and extended into MFG and PRG regions. In adults, the dorsal PRG extension was even stronger, resulting in higher convergence in the direct statistical comparison in adult compared with child studies. The anterior reading circuit in the left IFG was described in numerous reviews and meta‐analyses [Fiez and Petersen, 1998; Jobard et al., 2003; Price, 2012; Pugh et al., 2000; Richlan, 2012, 2014; Sandak et al., 2004; Taylor et al., 2013; Turkeltaub et al., 2002; Vigneau et al., 2006]. Its exact function, however, is still not clear [Richlan et al., 2014]. Functions associated with this region include linguistic processes such as grapheme–phoneme conversion [Jobard et al., 2003], phonological output computation [Taylor et al., 2013], lexical access [Heim et al., 2013], semantics [Binder and Desai, 2011], and speech planning and comprehension [Price, 2012], as well as nonlinguistic processes such as executive functions, affective, and interoceptive processes including working memory, reasoning, decision‐making, inhibition, attention, and emotion [Laird et al., 2011].

The present finding that the left IFG was identified not only in adult but also in child studies is in line with the results of the meta‐analysis on child reading by Houdé et al. [2010], which identified very similar peaks in left IFG and PRG regions. In developmental studies, the typical pattern is reliable left IFG engagement even in the early stages of reading acquisition [e.g., Church et al., 2008, Gaillard et al., 2003] and increase of engagement with increasing age [Bitan et al., 2007**b**; Booth et al., 2003, 2004; Brown et al., 2005; Schlaggar et al., 2002; Shaywitz et al., 2002; Turkeltaub et al., 2003]. Our meta‐analysis showed that the peak of the age‐related increase was not located in the IFG proper but in the most dorsal part of the left inferior frontal cluster corresponding to the PRG. Similarly, investigating reading‐related activation in a sample of children ranging from 9 to 15 years, the developmental fMRI study by Bitan et al. [2007b] identified a large left IFG cluster activated across all ages and a small left dorsal PRG cluster that showed an increase in activation with increasing age. The left dorsal PRG cluster was in close proximity (Euclidean distance 11 mm) to the local peak of our meta‐analytic finding of higher convergence in adult compared with child studies. Furthermore, our left PRG peak was very close (10 mm) to a peak associated with phonological processing in the meta‐analysis by Vigneau et al. [2006].

Interestingly, Koyama et al. [2011] found that resting‐state functional connectivity between the left PRG and other motor regions correlated positively with reading standard scores across children and adults. They interpreted the stronger motor coupling in better readers as reflecting automatized articulation. Recently, in line with the various functions associated with left IFG and PRG regions, it was shown that the PRG serves as a network hub, which can flexibly interact with other regions to support various cognitive processes required for linguistic as well as for nonlinguistic tasks [Power et al., 2013]. This interpretation is also supported by a similar resting‐state functional connectivity study by Vogel et al. [2013], who showed that reading engages largely domain‐general functional brain networks such as the fronto‐parietal and cingulo‐opercular control networks or the default mode network (see also Schurz et al., 2014b).

### Superior Temporal Gyrus

The left STG was identified with convergent reading‐related activation in children but not in adults. This finding received further support in the meta‐analytic difference map, where the left STG was shown to exhibit significantly higher convergence in child compared with adult studies. This finding is not surprising given the important role attributed to the left STG in early reading acquisition [Pugh et al., 2000; Sandak et al., 2004; Turkeltaub et al., 2003]. One may speculate, however, that the present finding of more consistent STG activation in children may be resulting from the higher proportion of studies that used rhyming tasks (10 child studies vs. 4 adult studies). To account for this, the overall match included a higher proportion of adult studies that used nonword stimuli and thus required phonological processing (3 child studies vs. 8 adult studies).

It was assumed that the left STG is essentially involved in phonology‐based reading processes (i.e., grapheme–phoneme conversion, phonological assembly), which are predominant in beginning readers [Jobard et al., 2003]. The left STG peak of our meta‐analytic finding of higher convergence in child compared with adult studies was in close proximity (Euclidean distance 9 mm) to a peak associated with phonological processing in the meta‐analysis by Vigneau et al. [2006]. Furthermore, our left STG peak was very close (6 mm) to a peak identified with decreasing activation with increasing age by Bitan et al. [2007b]. Age‐related decrease in left STG activation together with age‐related increase in left PRG activation was interpreted as reflecting a shift from reliance on sensory auditory representations to reliance on phonological segmentation and covert articulation, at least for performing rhyming judgment on visually presented words [Bitan et al., 2007b].

The left STG plays a major role in speech perception and production [Price, 2012] and serves as an interface between multimodal sensory and motor regions [Hickok and Poeppel, 2007]. Furthermore, it was shown that the left STG is concerned with the integration of auditory and visual information [Van Atteveldt et al., 2004], which is an essential part of successful reading acquisition. Both dyslexic children and dyslexic adults were found to exhibit left STG dysfunctions during letter‐speech sound integration [Blau et al., 2009, 2010]. Recently, combining multivoxel pattern analysis and functional and structural connectivity analysis, Boets et al. [2013] reported that the problem of dyslexic readers does not concern the integrity of stored phonetic representations in the left STG per se, but the access to these representations via left IFG regions.

As mentioned earlier, Paulesu et al. [2000] reported higher STG activation in readers of a shallow orthography (Italian) compared with readers of a deep orthography (English). This resemblance is hardly surprising given that both child readers and readers of a shallow orthography primarily rely on phonology‐based reading processes. Given that the proportion of shallow and deep orthography studies in the present meta‐analysis is approximately the same, the finding of higher convergence of STG activation in children reflects an age‐related change in reading strategies rather than a bias with respect to the writing systems of the included studies.

### Bilateral Supplementary Motor Area

The bilateral SMA was identified with convergent reading‐related activation in both children and adults. Convergence in child studies, however, was significantly higher than convergence in adult studies, with 17 and 13 studies contributing to the identification of the meta‐analytic clusters, respectively. Anatomically, the peak of the meta‐analytic difference map was located in an anterior part of the SMA, corresponding to the pre‐SMA and including the supplementary eye field (associated with generation and control of eye movements). Again, the identification of the SMA with reading‐related activation is in line with the meta‐analysis of child reading by Houdè et al. [2010] and with further meta‐analyses of adult reading [Fiez and Petersen, 1998; Price, 2012; Taylor et al., 2013; Turkeltaub et al., 2002].

In the domain of language, the function of the SMA was associated with overt articulation (motor execution), whereas the function of the more anterior located pre‐SMA was associated with covert articulatory planning for the production of speech sounds (sequencing motor plans) [Price, 2012]. In addition, the pre‐SMA was assumed an inhibitory role in the initiation of vocal and manual responses [Xue et al., 2008]. More generally, the SMA was assumed to be part of a control network that is involved in the stable maintenance of goal‐directed behavior over entire task epochs [Dosenbach et al., 2007, 2008]. Interestingly, our meta‐analyses identified bilateral SMA as well as left PPC regions, which are thought to form a fronto‐parietal control network involved in the switching between internally and externally focused goal‐directed cognition [Corbetta et al., 2008; Spreng et al., 2010].

### Limitations

Any meta‐analysis is limited by the characteristics of the original studies. In the case of age‐related meta‐analytic comparisons, specific limitations exist with respect to in‐scanner activation tasks and behavioral performance, neuroanatomical maturation, cerebral vasculature, and data acquisition and analysis protocols. In the present meta‐analysis, we attempted to match the child and adult studies as closely as possible. Highest priority was given to matching in‐scanner activation tasks and stimuli, because we assume that this has the biggest impact on reported brain activation, followed by native language, and sample size (see Materials and Methods section). In our final set of studies, children compared with adults exhibited lower accuracy (correct responses: 89% vs. 94%) and longer latencies (reaction times: 1600 ms vs. 1113 ms). This reflects the natural situation of higher reading‐related skills in adults. It is plausible to assume that the present meta‐analytic finding of higher convergence in child compared with adult studies in the bilateral SMA (part of the fronto‐parietal control network) is the consequence of this behavioral performance difference reflecting greater mental effort due to task difficulty in children.

It is important to note that a difference identified between two meta‐analytic maps does not mean that activation is higher in one group than in the other. Rather it means that the observed activation is more consistent across studies in one group than in the other. To identify differences in the activation strength the method of choice is a single study that directly compares activation in one group to activation in another group. Thus, single studies and meta‐analyses should complement each other to provide a holistic picture of functional neuroanatomical correlates of reading in children and adults.

Despite the limitations, the main strength of our approach is the unbiased and objective quantification of functional neuroanatomical commonalities and differences between child and adult reading. This study is the first direct comparison of reading‐related activation in children and adults within the same meta‐analytic framework. In contrast to narrative reviews, coordinate‐based meta‐analysis provides an unbiased synthesis of original studies and thus has the potential to detect previously overlooked findings. In addition, it results in precise specification of the neuroanatomical location of systematic effects.

## CONCLUSION

Using coordinate‐based meta‐analysis, this study provides an objective quantification of functional neuroanatomical commonalities and differences between children and adults during reading. Reading‐related activation common to children and adults was identified in left ventral OT and left IFG regions. Higher convergence in studies with children was found in the left STG and in the bilateral SMA. In contrast, higher convergence in studies with adults was found in bilateral posterior OT and left dorsal PRG regions. The pattern of results in the OT cortex was of specific interest, as it suggests an early engagement of left anterior and middle OT regions (corresponding to the VWFA) in beginning readers, whereas posterior OT regions exhibited specifically reading‐related recruitment only in more experienced readers. Likewise, the left dorsal PRG may serve as an important hub between functional networks relevant for reading primarily in adult readers, whereas the left IFG plays an important role in linguistic and nonlinguistic processes early on in reading acquisition.

## Supporting information

Supplementary Information
