# Supplementary material for: Reading in the brain of children and adults: A meta‐analysis of 40 functional magnetic resonance imaging studies
Source: Hum Brain Mapp. 2015 Jan 27;36(5):1963–81. doi: 10.1002/hbm.22749 (PMC4950303; doi:10.1002/hbm.22749)
Supplement: Supplementary file 1 — Supplementary Information [file HBM-36-1963-s001.docx]

**SI**. Supplementary information used for matching procedure of fMRI studies

|  |  |  |  |  |  |  |  |  |  |  |
| --- | --- | --- | --- | --- | --- | --- | --- | --- | --- | --- |
| Pair | Year | First author | Horizontal visual angle of stimuli | Stimulus details | MRI field strength (Tesla) | Smoothing kernel  FWHM (mm) | Analysis restricted to correct trials only | Analysis | Template used for normalization |  |
|  |  |  |  |  |  |  |  |  |  |  |
| *Children* | | | | | | | | | | |
| 1 | 2010 | Bach | n/a | 4 capital letters | 3 | 9 | no | random effects | MNI template |  |
| 2 | 2002 | Backes | n/a | n/a | 1.5 | 8 | no | random effects | n/a |  |
| 3 | 2006 | Bitan | n/a | n/a | 1.5 | 7 | no | random effects | T1 template volume MNI |  |
| 4 | 2007a | Bitan | n/a | 4-7 lower case letters,  1 syllable | 1.5 | 10 | no | random effects | T1 template volume MNI |  |
| 5 | 2007b | Bitan | similar for words & symbols | 4-7 lower case letters,  1 syllable | 1.5 | 10 | no | random effects | T1 template volume MNI |  |
| 6 | 2006 | Blumenfeld | n/a | lower case letters | 1.5 | 7 | no | random effects | T1 template volume MNI |  |
| 7 | 2001 | Booth | n/a | lower case letters | 1.5 | 7 | no | random effects | standard template |  |
| 8 | 2003 | Booth | n/a | lower case letters | 1.5 | 7 | no | random effects | T1 template volume MNI |  |
| 9 | 2007a | Booth | n/a | lower case letters | 1.5 | 10 | no | random effects | T1 template volume MNI |  |
| 10 | 2009 | Brem | 1.1°-3° | 4-12 letters | 1.5 | 9 | no | random effects | MNI template |  |
| 11 | 2006 | Cao | n/a | lower case letters | 1.5 | 10 | no | random effects | T1 template volume MNI |  |
| 12 | 2008 | Cao | n/a | lower case letters | 1.5 | 10 | no | random effects | T1 template volume MNI |  |
| 13 | 2001b | Gaillard | n/a | n/a | 1.5 | 9 | no | n/a | T1 template volume MNI |  |
| 14 | 2003 | Gaillard | n/a | sentence in 36 point Geneva font | 1.5 | 8 | no | fixed effects | T1 template volume MNI |  |
| 15 | 2006a | Hoeft | n/a | n/a | 3 | 8 | no | random effects | T1 template volume MNI |  |
| 16 | 2006b | Hoeft | n/a | n/a | 3 | 8 | no | random effects | T1 template volume MNI |  |
| 17 | 2007 | Hoeft | n/a | n/a | 3 | 8 | no | random effects | T1 template volume MNI |  |
| 18 | 2006 | Noble | n/a | 4 letters | 1.5 | 8 | no | random effects | T2 template volume MNI |  |
| 19 | 2009 | Rimrodt | n/a | sentence of 6 words | 1.5 | 8 | no | random effects | T1 template volume MNI |  |
| 20 | 2009 | Van der Mark | 2.2° (range: 1.3°-3°) | 4.5 (0.7) letters | 3 | 9 | yes | random effects | MNI template |  |
|  |  |  |  |  |  |  |  |  |  |  |
| **S1.** *Continued* | | | | | | | | | | |
| *Adults* | | | | | | | | | | |
| 1 | 2000 | Mechelli | n/a | 4-6 lower case letters, Courier New font | 2 | 8 | no | fixed effects | T2* template volume TC |  |
| 2 | 2002 | Cohen | 2°-6° | 3-6 lower case letters,  1-3 syllables | 1.5 | 8 | no | random effects | template volume MNI |  |
| 3 | 2005 | Bitan | n/a | n/a | 1.5 | 7 | no | random effects | T1 template volume MNI |  |
| 4 | 2002 | Booth | n/a | lower case letters,  1-2 syllables | 1.5 | 7 | no | random effects | n/a |  |
| 6 | 1999 | Kiehl | n/a | 3-4 capital letters | 1.5 | 8 | no | n/a | n/a |  |
| 5 | 2005 | Binder | 3.5° | 4-6 lower case letters, Geneva font | 1.5 | 7 | yes | random effects | n/a |  |
| 7 | 2001 | Booth | n/a | lower case letters | 1.5 | 7 | no | fixed effects | n/a |  |
| 8 | 2003 | Booth | n/a | lower case letters | 1.5 | 7 | no | random effects | T1 template volume MNI |  |
| 9 | 1999 | Chee | n/a | n/a | 1.5 | n/a | no | n/a | n/a |  |
| 10 | 2009 | Brem | 1.1°-3° | 4-12 letters | 1.5 | 9 | no | random effects | MNI template |  |
| 11 | 2005 | Burton | n/a | 3-5 letters | 1.5 | n/a | no | random effects | n/a |  |
| 12 | 2003 | Cohen | maximal 4° | 3-9 lower case letters, 1-4 syllables | 1.5 | 5 | no | random effects | template volume MNI |  |
| 13 | 2000 | Robertson | n/a | whole sentences | 3 | 5 | no | fixed effects | n/a |  |
| 14 | 2001 | Ferstl | n/a | sentences of 6.9 words | 3 | 4.2 | no | random effects | n/a |  |
| 15 | 2001 | Dehaene | 2.5° | 5 letters | 3 | 15 | no | random effects | n/a |  |
| 16 | 2006 | Binder | 2.7° | lower case Geneva font | 1.5 | 6 | no | random effects | n/a |  |
| 17 | 2001 | Poldrack | n/a | n/a | 1.5 | 6 | no | random effects | MNI305 template |  |
| 18 | 2000 | Tagaments | n/a | n/a | 1.5 | 10 | no | n/a | MNI EPI reference image |  |
| 19 | 2005 | Xu | 7.6° | lower case letters, Times New Roman font | 3 | 8 | no | random effects | T1 template volume MNI |  |
| 20 | 2004 | Rapp | n/a | sentences presented in  2 lines | 1.5 | 12 | no | random effects | T1 template volume MNI |  |

*Note*. MNI = Montreal Neurological Institute; TC = Talairach Coordinates
